# Assessing treatment adherence in psychiatry: a comparison of clinical judgment and therapeutic drug monitoring in acute care

**DOI:** 10.1097/YIC.0000000000000610

**Published:** 2026-03-25

**Authors:** Giorgio Pigato, Nicola Meda, Marco Paladin, Tommaso Toffanin, Carlo Artusi, Fabio Sambataro

**Affiliations:** a Azienda Ospedale-Università di Padova; b Department of Neuroscience (DNS), University of Padova, Padua, Italy

**Keywords:** acute setting, adherence, antidepressant, antipsychotic, bipolar disorder, major depressive disorder, mood stabilizer, personality disorder, schizophrenia spectrum disorder, therapeutic drug monitoring

## Abstract

Treatment adherence is the cornerstone of the management of psychiatric disorders in the prevention of acute symptom exacerbation, relapse, and hospitalization. Patients are usually treated with psychotropic polypharmacy, including complex regimens, which may contribute to nonadherence. In this real-world acute setting study, we used direct [therapeutic drug monitoring (TDM)] and indirect (emergency room and acute ward clinical judgments) measures to assess adherence in 145 patients with severe mental illnesses. Logistic regressions were used to predict adherence and agreement between adherence measures. The adherence estimates were predicted by sociodemographic and clinical factors and differed across methods: 24.8% for TDM, 55.9 and 50.4% for emergency room and acute ward clinical judgments, respectively. TDM aligned with clinical judgments in about 50% of cases, whereas the agreement between independent clinical judgments reached 84.9%. The concordance on adherence estimates between methods was influenced by sociodemographics, lifestyle, and clinical factors. Our findings underscore a discrepancy between different methods of adherence estimation. These methods capture distinct aspects of adherence and differ in their applicability and reliability, especially in acute settings. Future studies, integrating direct and indirect adherence estimates, are warranted to improve the accuracy of treatment adherence estimation for decision-making in managing exacerbation of psychiatric disorders.

## Introduction

Adherence refers to “the extent to which a person’s behavior – taking medication, following a diet, or executing lifestyle changes, corresponds with agreed recommendations from a healthcare professional” (Sabaté and World Health Organization, 2003). The lack of adherence to medication is a critical issue for the treatment of medical illness and can increase the risk of symptoms re-exacerbation and can explain up to one-quarter of all visits to the emergency room (Olshaker *et al.*, 1999), with mental illness representing the largest part of these visits (almost 30%), with an odds ratio (OR) of 22.74 (Heaton *et al.*, 2013). Scarce adherence to psychopharmacological treatment results in frequent relapses and a higher risk of hospitalization for mental disorders (Gilmer *et al*., 2004; Lang *et al*., 2010) and can hamper the decision-making process for treatment prescription. The overall rate of psychotropic medication nonadherence is estimated at 49%, ranging from 44% in bipolar disorder (BPD) and 50% in major depressive disorder (MDD) to 56% in schizophrenia spectrum disorders (SSD) (Semahegn *et al.*, 2020).

Medication-related factors play a great role in scarce adherence among severe mental illnesses (SMI), almost doubling this risk relative to other preventable causes (Fung *et al*., 2019; Pedrosa-Naudín *et al.*, 2022; Guo *et al.*, 2023) and could be addressed by clinicians. Psychotropic polypharmacy, which is defined as the use of two or more psychotropic medications in the same patient (Sarkar, 2017), represents a critical situation, especially when involving complex regimens (the use of ≥4 medications) (Weinstock *et al.*, 2014; Gaudiano *et al*., 2018). The prevalence of complex psychotropic polypharmacy varies widely across diagnoses, ranging from 13 to 90% (Stahl, 2002; De Las Cuevas and Sanz, 2004; Tungaraza *et al.*, 2010). Specifically, it is estimated to be about 20% in SSD (Gallego *et al.*, 2012), between 36% (Greil *et al.*, 2012) and 85% (Fornaro *et al*., 2016) in BPD, and is approximately 23% in MDD (Rhee and Rosenheck, 2019).

Psychotropic treatment adherence evaluation remains challenging (Kim *et al.*, 2021). It can be assessed with direct [i.e. directly observed therapy, therapeutic drug monitoring (TDM)] or indirect methods. The most accurate estimation method is TDM, which consists of comparing the expected levels of medication given the prescription dosage with direct measurement of serum/plasma drug concentration in a steady state at the time of minimal drug concentrations (Hiemke *et al*., 2018). TDM estimation, which requires expertise and a well-equipped laboratory, is accurate for monotherapy and can become challenging for the polypharmacy of complex regimens (Hiemke *et al*., 2018), especially in acute psychiatric settings. Notably, in this context, a single drug outside the expected plasma levels may reflect a deliberate clinical adjustment (targeted to a specific symptom) or arise from a medical issue, rather than true patient noncompliance. Furthermore, a strict binary classification does not account for the relative importance of different psychotropic agents, potentially leading to an overestimation of nonadherence. In particular, limited guidelines, drug–drug interactions, comorbidities, patients’ uncooperativeness, and time constraints for rapid decision-making contribute to the complexity of the interpretation (Liang *et al.*, 2024).

Indirect approaches include objective or subjective methods. Although indirect methods are widely used in everyday clinical practice for their feasibility, low cost, and invasiveness, they are less sensitive and generalizable, and their reliability has been questioned (Velligan *et al.*, 2017). Only a limited number of studies in the literature have investigated their agreement with objective methods in mental disorders, and a few have been conducted in acute settings (Lopez *et al.*, 2017; Geretsegger *et al*., 2019; Brasso *et al.*, 2021). A low concordance between indirect (i.e. clinical judgment in the emergency room, and self-rated adherence rating scales at the psychiatric ward) and direct measures (TDM) of adherence in the acute setting has been consistently reported (Lopez *et al.*, 2017; Geretsegger *et al*., 2019; Brasso *et al.*, 2021). The use of two indirect assessments could increase the reliability of clinical judgments by minimizing single-source bias, capturing additional information, and increasing confidence of the results (Lehmann *et al*., 2014); however, this approach remains relatively unexplored.

**Fig. 1:**
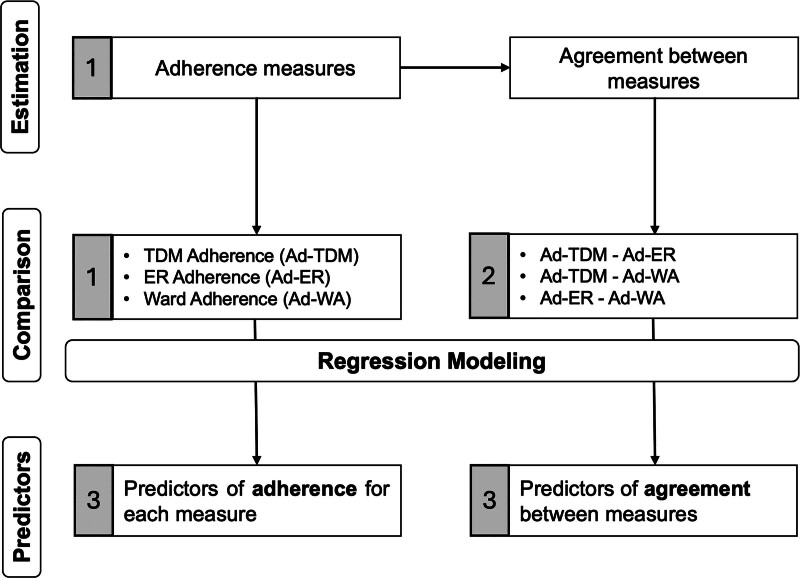
Conceptual framework illustrating the study aims and analytical pipeline. The framework of our study comprised these components (on the left): *Estimation* of individual adherence measures using different methods and their pairwise agreement rates. *Comparison* of different adherence measure estimates, Ad-TDM, Ad-ER, and Ad-WA, as well as the agreement rates between them, and identification of *Predictors* of both adherence and agreement rates using regression modeling. The aims of this study are indicated with numbers in gray boxes: (a) to evaluate medication adherence; (b) to compare the agreement between the adherence estimation methods; and (c) to identify factors influencing the reliability of adherence measurement and agreement. Ad-ER, adherence defined emergency room; Ad-TDM, adherence defined therapeutic drug monitoring; Ad-WA, adherence defined ward assessments.

In the present study, we sought to evaluate medication adherence with multiple measurements in patients with SMI who presented to the emergency room department and were later admitted to the psychiatric ward. To increase the reliability and ecological validity of the estimates, we evaluated medication adherence for stable treatments, including multiple drugs, and used direct (TDM) as well as two longitudinal indirect assessments (emergency room and acute ward psychiatrist’s judgment). We compared the agreement between estimation methods and identified factors influencing the reliability of medication adherence and measurement agreement (Fig. 1).

We hypothesized that treatment adherence in SMI would be limited, regardless of the estimation methods, and that the level of agreement between methods would be low.

## Materials and methods

### Study cohort

The present work was conducted at the Acute Psychiatric Ward of the “Azienda Ospedale-Università di Padova”, University Hospital of Padua, Italy. Patients enrolled in our study were consecutively admitted to the Psychiatric Ward after an urgent psychiatric consultation at the emergency room department of the same hospital (see Table 1 for demographics and clinical information). Inclusion criteria were the following: (a) age older than 18 years; (b) having a diagnosis of an SMI, including SSD, BPD, MDD, and personality disorders (Evans *et al.*, 2016), (c) being currently on greater than or equal to 1 psychiatric medication taken at steady state (i.e. time from the first prescription to emergency room consultation greater than or equal to five times of half-life elimination of the drug), the plasma levels of which were doseable with TDM. Exclusion criteria were (a) inability to provide valid consent; (b) diagnosis of major neurocognitive disorders (i.e. delirium or dementia); (c) being on long-acting antipsychotic treatment; (d) drug intoxication. In this study, we considered each admission to the ward as a statistical unit, given that each admission might be related to treatment nonadherence (e.g. a patient might be medication-adherent at the first admission and nonadherent at the subsequent ones). For convenience, throughout the manuscript, we will use the term patient instead of hospitalization. All patients included in this study previously signed a written informed consent (or legal guardian consent) for the general use of their data for research purposes, anonymously and in aggregate form, according to our local internal review board. This research was performed following the Helsinki Declaration of 1975 guidelines.

**Table 1:** Demographic characteristics of the sample and breakdown by diagnosis

Characteristics	Overall (*n* = 145)	PD (*n* = 29)	SSD (*n* = 55)	BPD (*n* = 39)	MDD (*n* = 22)	*P* value
Age, mean (SD)	48.23 (14.55)	48.41 (13.53)	44.85 (14.40)	50.92 (15.41)	51.64 (13.75)	0.14
Sex (female), *n* (%)	64 (44.1)	9 (31.0)	27 (49.1)	21 (53.8)	7 (31.8)	0.14
Years of education (>8 years), *N* (%)	81 (55.9)	13 (44.8)	27 (49.1)	27 (69.2)	14 (63.6)	0.12
Marital status (in a relationship), *N* (%)	38 (26.2)	6 (20.7)	8 (14.5)	15 (38.5)	9 (40.9)	0.02^a^
Working condition, employed (%)	59 (40.7)	11 (37.9)	15 (27.3)	19 (48.7)	14 (63.6)	0.02^a^
Living situation, *N* (%)						
With family	81 (55.9)	17 (58.6)	28 (50.9)	22 (56.4)	14 (63.6)	0.26
Alone	48 (33.1)	10 (34.5)	16 (29.1)	15 (38.5)	7 (31.8)	
Therapeutic community	16 (11.0)	2 (6.9)	11 (20.0)	2 (5.1)	1 (4.5)	
Age group (years), *N* (%)						
18–39	37 (25.5)	7 (24.1)	20 (36.4)	8 (20.5)	2 (9.1)	0.20
40–59	79 (54.5)	18 (62.1)	25 (45.5)	21 (53.8)	15 (68.2)	
60–99	29 (20.0)	4 (13.8)	10 (18.2)	10 (25.6)	5 (22.7)	
Compulsory admission, *N* (%)	11 (7.58)	3 (10.3)	5 (9.1)	3 (7.7)	0	0.36
Duration of illness (years), mean (SD)	15.88 (15.52)	9.03 (11.78)	18.29 (16.52)	18.72 (16.75)	13.86 (12.57)	0.03^a^
CGI – severity, mean (SD)	5.08 (0.98)	4.78 (0.95)	5.29 (0.94)	5.11 (1.16)	4.91 (0.75)	0.18
Substance use disorder, *N* (%)	18 (12.4)	7 (24.1)	3 (5.5)	7 (17.9)	1 (4.5)	0.04
Alcohol use disorder, *N* (%)	19 (13.1)	8 (27.6)	2 (3.6)	5 (12.8)	4 (18.2)	0.02
No. cigarettes per day, *N* (%)						
0–10	86 (59.3)	15 (51.7)	36 (65.5)	21 (53.8)	14 (63.6)	0.46
>10	22 (15.2)	4 (13.8)	8 (14.5)	5 (12.8)	5 (22.7)	
>20	37 (25.5)	10 (34.5)	11 (20.0)	13 (33.3)	3 (13.6)	
Complex polypharmacy – psych (>4 drugs), *N* (%)	25 (17.2)	8 (27.6)	8 (14.5)	3 (7.7)	6 (27.3)	0.09

*P* value refers to the effect of diagnosis.

BPD, bipolar disorders; CGI, Clinical Global Impression; MDD, major depressive disorders; PD, personality disorders; Polypharmacy – psych, prescription of more than four psychotropic medications; SSD, schizophrenia-spectrum disorders; TDM, therapeutic drug monitoring.

a Not significant after Bonferroni correction for multiple comparisons.

### Clinical measures

For each patient, we collected demographic and clinical data, including education level, marital status, living situation, working condition, psychiatric diagnosis, duration of the illness, substance use disorder (SUD) or alcohol use disorder (AUD), medical comorbidity, smoking status, and current voluntary or compulsory admission to the psychiatric ward. Within the first day after admission, the Clinical Global Impression Severity Scale (CGI-S) was administered.

Psychiatric judgment on medication adherence, which was operationalized as a dichotomous answer (adherent: yes/no), was expressed immediately after the consultation in the emergency room [adherence assessed in the emergency room (Ad-ER)] and within 1 day from admission to the psychiatric ward by a second clinician [adherence assessed in the ward (Ad-WA)], who was a fully trained psychiatrist. The clinicians were blind to each other’s adherence judgment and TDM assessment. All clinicians underwent training sessions on the use and administration of these instruments to achieve reliability between 0.80 and 0.90 with yearly recalibration (Pigato *et al*., 2023).

### Therapeutic drug monitoring

Within 24 h after admission to the ward, the patients underwent blood sampling for routine tests and TDM assessment of all the following drugs and active metabolites: (a) antipsychotics: aripiprazole/dehydroaripiprazole, clozapine/norclozapine, haloperidol, lurasidone, olanzapine/N-desmetil-olanzapine, quetiapine/norquetiapine, risperidone/9-OH-risperidone, paliperidone, lurasidone; (b) antidepressants: citalopram/desmetil-citalopram, clomipramine, escitalopram/desmethyl-escitalopram, fluoxetine/desmethyl-fluoxetine, mirtazapine, sertraline/N-desmetil-sertraline, venlafaxine/desvenlafaxine; (c) mood stabilizers (or anticonvulsants): carbamazepine, gabapentin, lamotrigine, lithium, oxcarbazepine, and valproic acid.

HPLC coupled with tandem mass spectrometry (HPLC-MS/MS) was used to assess serum drug concentration. The concentration of lithium was measured with ion-selective electrodes. The actual to expected plasma level of medication, based on the daily dose of the individual patient, according to the Consensus Guidelines for Therapeutic Drug Monitoring in Neuro-Psychopharmacology 2017 (Hiemke *et al*., 2018), was estimated for each medication. Then, the dose-related reference range (DRRR), which is the expected range of a drug when administered at a given dose, was calculated for each drug.

TDM-defined adherence (Ad-TDM) to medications was assessed by the following, a priori, narrowed method of estimation, as in previous reports (Smith *et al*., 2020): (a) if the drug level (or its metabolite levels) was out of the expected range of DRRR (above or below), the patient was defined as nonadherent; (b) in the case of complex polypharmacy (defined as a patient taking ≥4 drugs), the patient was considered nonadherent when a mismatch between DRRR and observed levels for greater than or equal to 1 drug was observed. For instance, if a patient was prescribed one antidepressant, one mood stabilizer, and one antipsychotic drug, the mismatch for just one medication was sufficient to deem a patient nonadherent to drug treatment. To avoid overestimation of nonadherence when using TDM in polypharmacy, we carefully reviewed each patient’s record based on the clinical relevance of each drug within the primary diagnostic indication and drug–drug interactions as outlined in the 2017 guidelines of the TDM group of the Arbeitsgemeinschaft für Neuropsychopharmakologie und Pharmakopsychiatrie (Hiemke *et al*., 2018). In these guidelines, while acknowledging that alcohol or drug of abuse in AUD/SUD can affect TDM, no specific recommendations are provided. Therefore, specific adjustments were not applied in our study. Moreover, drugs prescribed at subtherapeutic doses relative to the recommended range for the primary diagnosis were excluded from the estimation of nonadherence. To investigate the specificity of the relationship between TDM estimated adherence and the main diagnosis, as in a previous report (Geretsegger *et al*., 2019), we performed a subanalysis of TDM adherence for the drug class (antipsychotics, antidepressants, and mood stabilizers) that was associated with the main diagnosis. Specifically, adherence was limited to antipsychotics for SSD, mood stabilizers for BPD, and antidepressants for MDD, respectively. Personality disorders were excluded from this subanalysis because of the lack of specific indications for psychopharmacotherapy.

### Statistical analysis

Data from an anonymized spreadsheet were curated using R Studio 2021.09.1 (R version 4.1.2) (R Core Team, 2019), and descriptive statistics were computed for each measure. Demographic differences were tested with *t* test/one-way analysis of variance in the case of continuous variables, with Tukey’s post-hoc test when appropriate, or *χ*^2^ test for frequency tables, respectively. Differences in agreement between adherence measures (Ad-TDM, Ad-ER, and Ad-WA) according to diagnosis (as reported in Table 2) were computed with the *χ*^2^ test. To estimate the model of medication adherence for each measure, we defined several binomial regression models, which sought to identify variables significantly associated with drug adherence or nonadherence. Each *k* regression model performance was then head-to-head compared to a *k* + 1 regression model, where the *k* + 1 model is composed of the same variables as the *k* model, except for an additional independent variable. Model performance was compared using the *anova* function [lme4 package (Bates *et al.*, 2019)], and the model with the lowest Bayesian Information Criterion (BIC) was selected. Finally, each independent variable’s weight in changing the odds of adherence to medication was evaluated through the estimation of *β* regression coefficients. A backward–forward stepwise approach was applied for variable selection: the contribution of each relevant independent variable to the model was evaluated, and only the variables with a statistically significant effect size that improved the model (i.e. reduced the BIC) were maintained in the regression model. In these models, civil status was used as a random factor, as it showed no significant association with any of the outcomes of interest in our sample. Also, marital status was modeled as a random effects variable to capture between-subject variability related to stable social and living conditions, potentially influencing psychiatric outcomes. We applied this approach to evaluate which variables were significantly associated with a higher likelihood of adherence according to each measure (Ad-TDM, Ad-ER, and Ad-WA), as well as with the likelihood of agreement between each adherence measure.

**Table 2:** Prevalence of treatment adherence according to measurement method and agreement rates among measures

Measures	Overall (*n* = 145)	PD (*n* = 29)	SSD (*n* = 55)	BPD (*n* = 39)	MDD (*n* = 22)	*P* value
TDM adherence (Ad-TDM), *N* (%)	36 (24.8)	3 (10.3)	20 (36.4)	9 (23.1)	4 (18.2)	0.05
ER adherence (Ad-ER), *N* (%)	81 (55.9)	12 (41.4)	32 (58.2)	21 (53.8)	16 (72.7)	0.15
Ward adherence (Ad-WA), *N* (%)	73 (50.4)	11 (37.9)	29 (52.7)	21 (53.8)	12 (54.5)	0.52
Agreement Ad-WA – Ad-ER, *N* (%)	123 (84.9)	24 (82.8)	48 (87.3)	33 (84.6)	18 (81.8)	0.91
Agreement Ad-ER – Ad-TDM, *N* (%)	68 (46.9)	18 (62.1)	25 (45.5)	17 (43.6)	8 (36.4)	0.27
Agreement Ad-WA – Ad-TDM, *N* (%)	76 (52.5)	12 (41.4)	27 (49.1)	18 (46.2)	12 (54.5)	0.81
TDM adherence for main drug indication, *N* (%)	71 (62.0)	N/A	41 (74.5)	16 (41.0)	12 (54.0)	0.004

*P* value refers to the effect of diagnosis.

Ad-ER, adherence defined emergency room; Ad-WA, adherence defined ward assessment; BPD, bipolar disorder; MDD, major depressive disorder; PD, personality disorders; SSD, schizophrenia-spectrum disorder; TDM, therapeutic drug monitoring.

Data visualization was produced using the *ggplot2* (Wickham, 2009) R package; the lme4 (Bates *et al.*, 2019) package was used for regression modeling, while the model selection was guided by the work of Raftery (1995).

## Results

We collected demographic and clinical data on 145 patients (55.9% females, mean age 48.2 ± 14.5 years) of 128 patients (nine patients were admitted twice, two were admitted three times, 1 five times). Hereafter, the mean and SDs of demographic characteristics will refer to the description of the sample of 145 patients (Table 1). Patients were grouped into four diagnostic categories according to the Diagnostic and Statistical Manual of Mental Disorders, 5^th^ Edition: personality disorder (*n* = 29), SSD (*n* = 55), BPD (*n* = 39), and MDD (*n* = 22). No statistically significant differences were found in age, years of education, marital status, unemployment, living situation, compulsory admission to the ward, number of cigarettes per day, or polypharmacy, and clinical severity assessed using CGI-S when stratifying by diagnoses or sex. For the duration of the illness, there was a statistically significant difference for diagnosis (*F* = 3.005, *df* = 3, *P* = 0.03), with a longer duration for SSD compared with personality disorder [mean difference = 9.26 years, 95% confidence interval (CI): 0.18–18.33, *P* = 0.043]. Furthermore, in terms of substance use, there was a significant difference in the frequency of SUD [*χ*^2^ (3143) = 8.4, *P* = 0.037] and AUD [*χ*^2^ (3143) = 10.17, *P* = 0.017] between diagnoses.

The mean adherence estimates were 24.8% for Ad-TDM, 55.9% for Ad-ER, and 50.3% for Ad-WA (Table 2). Although we considered the possibility of the TDM estimates being higher than expected, none of the patients had drug levels above the expected range.

The Ad-TDM rates were 36.4% for SSD, 23.1% for BPD, 18.2% for MDD, and 10.3% for personality disorder; the Ad-ER rate was 58.2% for SSD, 53.8% for BPD, 72.7 for MDD, and 14.4% for personality disorder; and the Ad-WA rate was 52.7% for SSD, 53.8% for BPD, 54.5% for MDD, and 37.9% for personality disorder, respectively. The Ad-TDM rates specific per diagnostic groups for the main indications were 74.5% for antipsychotics in SSD, 41.0% for mood stabilizers in BPD, and 54.0% for antidepressants in MDD, respectively.

The best model that predicted Ad-TDM included the group and duration of the illness (marginal *R*^2^ = 0.14; Table 3). The odds of adherence showed an almost four-fold mean decrease in the 40–59 age group compared with the younger group (OR = 0.26, 95% CI: 0.10–0.64, *P* < 0.01), and a 3% increase for each year of illness (OR = 1.03, 95% CI: 1.00–1.05, *P* < 0.05).

**Table 3:** Factors predicting adherence with adherence methods

Measures	TDM adherence (Ad-TDM)	WA adherence (Ad-WA)	ER adherence (Ad-ER)
Predictors	OR (95% CI)	OR (95% CI)	OR (95% CI)
(Intercept)	0.47 (0.21–1.03)	0.10 (0.01–0.94)*	0.04 (0.00–0.41)**
Age group: 40–59 (ref: age group: <40)	0.26 (0.10–0.64)**	–	–
Age group: 60–99	0.40 (0.13–1.21)	–	–
Duration of Illness (years)	1.03 (1.00–1.05)*	–	–
AUD (ref: no AUD)	–	0.23 (0.06–0.93)*	0.18 (0.04–0.73)*
BMI (kg/m^2^)	–	1.10 (1.02–1.19)**	1.15 (1.05–1.25)**
Education: >12 years (ref: ≤12 years)	–	0.42 (0.17–1.05)	–
Employed (ref: unemployed)	–	2.48 (1.00–6.15)	–
* *Marginal *R*^2^	0.140	0.226	0.245

Significant predictors from a multiple regression model are indicated in the leftmost column.

The reference group for the predictor is reported in the first column.

* *P* < 0.05;

** *P* < 0.01.

Ad-ER, clinician judgment adherence in the emergency room; Ad-TDM, adherence defined by therapeutic drug monitoring; Ad-WA, clinician judgment adherence in the acute ward; AUD, alcohol use disorder; CI, confidence interval; OR, odds ratio.

The prediction model for Ad-ER (model marginal *R*^2^ = 0.24) showed increased adherence with higher BMI (OR = 1.15, 95% CI: 1.05–1.25, *P* < 0.01) and reduced by a history of AUD (OR = 0.18, 95% CI: 0.04–0.73, *P* < 0.05; Table 3).

Ad-WA was predicted by a higher BMI (with an increase of 10% in the odds of adherence for each BMI point: (OR = 1.10, 95% CI: 1.02–1.19, *P* < 0.01) and reduced by a history of AUD (with an almost four-fold mean decrease, OR = 0.23, 95% CI: 0.06 – 0.93, *P* < 0.05; Table 3).

### Agreement between adherence assessed by therapeutic drug monitoring, adherence-assessed in the emergency room, and adherence assessed in the ward

Ad-TDM and Ad-ER agreed for 46.9% of the patients (Table 3). The agreement between the Ad-TDM and Ad-ER models had a marginal *R*^2^ = 0.24 (Table 3). The agreement rate between these measures was significantly reduced in patients with SSD (OR = 0.34, 95% CI: 0.12–0.96, *P* < 0.05 relative to personality disorder), with stable employment (70% reduction; OR = 0.29, 95% CI: 0.13–0.65, *P* < 0.01), and heavy smoking (>20 cigarettes per day OR = 0.37, 95% CI: 0.15–0.92, *P* < 0.05, relative to <10 cigarettes per day). On the other hand, the odds of agreement increased 3.5-fold in patients older than 60 years compared to those younger than 40 years (OR = 3.55, 95% CI: 1.15–10.9, *P* < 0.05; model marginal *R*^2^ = 0.24; Table 4). None of the other variables influenced the agreement rate.

**Table 4:** Factors predicting agreement between adherence estimation methods

Measures	Ad-TDM – Ad-ER agreement	Ad-TDM – Ad-WA agreement	Ad-ER – Ad-WA agreement
Agreement (%)	46.9	52.5	84.9
Predictors	OR (95% CI)	OR (95% CI)	OR (95% CI)
Intercept	2.83 (0.89–8.95)	1.46 (0.95–2.24)	17.50 (4.21–72.76)***
Age group: 40–59 (ref: age group: <40)	1.32 (0.54–3.20)	–	0.21 (0.05–0.96)*
Age group: 60–99	3.55 (1.15–10.99)*	–	0.50 (0.08–3.18)
Employed (ref: unemployed)	0.29 (0.13–0.65)**	0.50 (0.26–0.99)*	–
No. of cigarettes > 20/day (ref: 0–10)	0.37 (0.15–0.92)*	–	–
No. of cigarettes = 11–20/day	1.19 (0.43–3.36)	–	–
SSD (Ref: axis II Dis.)	0.34 (0.12–0.96)*	–	–
BPD	0.39 (0.13–1.20)	–	–
MDD	0.27 (0.07–1.02)	–	–
AUD (ref: no AUD)	–	–	–
* *Marginal *R*^2^	0.239	0.033	0.121

Agreement rate between adherence measures (computed as the rate of concordance, both adherent–adherent and not adherent–not adherent, between two measures). Significant predictors from a multiple regression model are indicated in the leftmost column.

The reference group for the predictor is reported in the first column.

* *P* < 0.05;

** *P* < 0.01;

*** *P* < 0.001.

Ad-ER, clinician judgment adherence in the emergency room; Ad-WA, clinician judgment adherence in the acute ward; AUD, alcohol use disorder; BPD, bipolar disorder; CI, confidence interval; MDD, major depressive disorder; OR, odds ratio; SSD, schizophrenia-spectrum disorder.

The agreement between Ad-TDM and Ad-WA was 52.5% (Table 3). The agreement between the Ad-TDM and Ad-WA model had a marginal *R*^2^ = 0.03 (Table 3). In this case, the agreement was reduced only by employment (50% reduction, OR = 0.50, 95% CI: 0.26–0.99, *P* < 0.05 relative to unemployment).

The agreement rate between Ad-ER and Ad-WA was 84.9% (Table 3). The agreement between the Ad-ER and Ad-WA models had a marginal *R*^2^ = 0.12 (Table 3). The odds of agreement were reduced for the 40–59 age group (OR = 0.21, 95% CI: 0.05–0.96, *P* < 0.05 relative to the <40 age group).

## Discussion

The assessment of psychotropic drug adherence is a crucial issue in the management of acute patients with SMI, and yet it has been relatively little investigated in the literature (Table 5). In our study, all adherence estimation methods consistently reported that patients with SMI had a low adherence to psychotropic medications at the time of hospital admission after an acute emergency room consultation, with the TDM estimated adherence being half of that expected by clinicians (Ad-ER and Ad-WA). Second, several factors, including age, illness duration, AUD, and BMI, were associated with drug adherence. Third, the agreement between Ad-TDM and Ad-ER, Ad-WA was lower in SSD, in those with stable employment, and in heavy smokers, while it was higher in older age. Employment also reduced the agreement between Ad-TDM and Ad-WA evaluations, whereas age was the only factor influencing the consistency between Ad-ER, Ad-WA. Fourth, Ad-TDM for the main diagnostic indication was greater compared with the overall Ad-TDM.

**Table 5:** Comparison of sample, design, and adherence estimates across previous studies and the current one

Measures	Present study	Lopez *et al*. (2017)	Geretsegger *et al*. (2019)	Brasso *et al*. (2021)
Sample (*N*)	145	97	161	133
Diagnosis (*N*, %)				
SSD	55 (37.9%)	71 (73.2%)	55 (34.2%)	57 (42.9%)
BPD	39 (26.9%)	26 (26.8%)	60 (37.3%)	76 (57.1%)
MDD	22 (15.2%)			
PD	29 (20.0%)		9 (5.6%)	
Anxiety disorder			25 (15.5%)	
Other			12 (7.4%)	
Drug regimen (*N*, %)				
1 Medication	15 (10.3%)	N/A	72 (44.7%)	16 (12%)
2–4 Medications	105 (72.4%)	N/A	89 (55.3%)	117 (87.9%)^a^
>4 Medications	25 (17.2%)	N/A	None	N/A
TDM adherence estimation				
Number of medications	≥1	1	1	1
Psychotropic drug class (number)	AP (all), MS (all), AD (all)	AP (1)	AP (1) or AD (1)	AP (1) or MS (1)
Transdiagnostic assessment	Yes	No	No	No
Drug diagnostic indication	Yes	No	Yes	Yes
Steady state	Yes	No	No	No
DRRR	Yes	No	No	Yes
Indirect measures of adherence estimation				
Type	Ad-ER, Ad-WA	Ad-ER	None	Self-report
Results				
Adherence-TDM by drug for the main diagnostic indication				
Overall, *N* (%)	36 (24.8%)	33 (34.0%)	44 (27.0%)	71 (53.3%)
SSD	41 (74.5%)	–	17 (30.9%)	29 (50.9%)
BPD	16 (41.0%)	–	12 (20.0%)	42 (55.3%)
MDD	12 (54.0%)	–		–
Adherence – clinical judgment in the ER				
Overall, *N* (%)	81 (55.9%)	57 (54.6%)	–	–
SSD	32 (58.2%)	41 (57.7%)	–	–
BPD	21 (53.8%)	12 (46.2%)	–	–
MDD	16 (72.7%)	–	–	–
PD	12 (41.4%)	–	–	–

AD, antidepressants; Ad-ER, clinician judgment adherence in the emergency room; Ad-WA, clinician judgment adherence in the acute ward; AP, antipsychotics; BPD, bipolar disorder; DRRR, dose-related reference range; MDD, major depressive disorder; MS, mood stabilizer; PD, personality disorder; SSD, schizophrenia-spectrum disorder; TDM, therapeutic drug monitoring.

a More than one medication.

Overall adherence defined by TDM was 24.8%, which is in line with two previous reports [27 and 34% in Geretsegger *et al*. (2019) and Lopez *et al.* (2017), respectively] but not with Brasso *et al*. (2021), who estimated a 53.3% adherence rate. Clinical judgment estimated an adherence of 55.9% for Ad-ER with rates of 58.2 and 53.8% for SSD and BPD, respectively. These results are in line with a previous report by Lopez *et al*. (2017), which estimated an overall adherence rate of 54.6%, 57.7% for SSD, and 46.1% for BP. The clinical judgment adherence rates at Ad-WA were 50.4% overall, 52.7% for SSD, and 53.8% for BPD. These rates are at Ad-WA in agreement with those at Ad-ER and comparable to those reported by Lopez *et al*. (2017)

The adherence defined by TDM varied by age and duration of the disease. Patients aged 40–59 were three times more likely not to adhere. In contrast with our results, an earlier study reported that extreme age groups are more likely to be nonadherent (Smilowitz *et al.*, 2020); however, methodological differences relative to the setting (e.g. private practice vs. ward/emergency room), the estimates of adherence (e.g. indirect measures vs. TDM), and the type of prescription (e.g. monotherapy vs. polypharmacy) may explain this discrepancy. Consistent with previous literature in patients with SSD, we found that a longer duration of the illness was associated with greater adherence to antipsychotic medications, thus extending this result to an acute hospital setting (Linden *et al*., 2001).

Finally, when comparing adherence for drug classes specific to the main indication, we found that the TDM estimated adherence rates were two-fold for SSD (74.5 vs. 36.4%, antipsychotics) and BPD (41.0 vs. 23.1%, mood stabilizers), and three-fold for MDD (54.0 vs. 18.2%, antidepressants) relative to overall TDM estimates for diagnosis. This result suggests that patients tend to be more adherent to the drug prescribed for their main diagnosis, which is essential in the evaluation of adherence. Treatment adherence is a more global phenomenon that goes beyond the main treatment for the indication, and the failure to follow a polypharmacy regimen can result in increased risk of relapse. For example, the combination of a mood stabilizer and an antipsychotic drug significantly reduces the risk of rehospitalization in SSD (Puranen *et al.*, 2023) as well as in BPD (Hochman *et al.*, 2016). Overall, adherence can be conceptualized as being context-dependent and not solely drug-dependent, thus suggesting that both TDM estimates are necessary for a comprehensive assessment.

For clinician-based judgments, both in the emergency room and acute ward settings, estimated drug adherence was influenced by alcohol use and body mass. In our study, comorbidity with AUD was associated with a 3–4-fold reduction in medication adherence estimates. Previous studies have shown a transdiagnostic negative effect of alcohol use on medication adherence (Drake *et al.*, 1989; Kashner *et al.*, 1991; Owen *et al.*, 1996; Verdoux *et al*., 2000). Emotional dysregulation, impulsivity, and novelty-seeking traits may underlie AUD and independently contribute to medication nonadherence (Liraud and Verdoux, 2000). A higher BMI was associated with a 10–15% increased likelihood of estimated adherence. Previous studies using electronic health records and medication order data identified BMI increase in the first months of antipsychotic treatment as suggestive of treatment compliance in outpatients with BPD and schizophrenia relative to other psychiatric diagnoses (Perkins *et al*., 2023). Unfortunately, we did not measure early changes in BMI and could not ascertain the direct contribution of this variable to the clinical judgment. Given the well-known weight gain increase due to the majority of psychotropic medications, we can reasonably hypothesize that clinician-based judgment on adherence could be influenced by body weight perception (Huizinga *et al.*, 2010). Indeed, subjective adherence measures are intrinsically influenced by cognitive biases and diagnostic stereotypes (Tversky and Kahneman, 1974). Indeed, clinicians may rely on salient clinical cues (Graber *et al.*, 2005), such as weight, employment status, or diagnosis, including AUD, which can bias their judgment of adherence, thus assuming treatment compliance even when these associations are not necessarily causal. Our findings of reduced agreement between subjective and TDM-related adherence assessments suggest that in clinical situations where objective measures are lacking, the estimation of adherence depends on clinicians’ heuristics rather than actual medication use patterns. Thus, sociodemographic and clinical features, including high BMI, AUD, or unemployment, may serve as warning signals for potential misjudgment of subjective adherence, thereby prompting a more careful evaluation and, when available, the complementary use of TDM assessment.

Our third goal was to identify factors that could affect clinicians’ judgment in the assessment of drug adherence while using TDM-defined adherence as a reference. The agreement rate between emergency room judgments and TDM was 46.9%. A diagnosis of SSD, high smoking, and occupational status significantly reduced the correspondence between these methods. The agreement rate between acute ward judgments and TDM was 52.5%. Being employed reduced the level of agreement. Diagnosis and sociodemographic factors are traditionally associated with clinicians’ expectations with regard to patient compliance with treatment and, therefore, may affect clinician estimation predominantly in the emergency room setting, where the information, if any, is less reliable. Regarding clinical judgment measures, the emergency room and ward psychiatrists agreed 84.9% of the time with respect to the assessment of medication adherence. The middle-aged group (40–59 years) showed reduced agreement between clinicians in adherence (about 80% reduction), and this is consistent with the finding of reduced TDM estimated adherence in these subjects.

Overall, we found a discrepancy between TDM and indirect measures. This result was expected. TDM measures capture a “snapshot” of drug levels at a specific time point without taking into consideration all the concurrent factors that may influence the pharmacokinetics of the drug, including patient-related factors. Conversely, indirect measures reflect an empirical evaluation of the global clinical presentation (e.g. clinical symptom profile, family and context-related sources, and health records, including medical history, previous diagnosis and treatments, etc.) without a direct assessment of drug levels (Nieuwlaat *et al*., 2014).

Regarding the TDM approaches employed in our study and those referenced above, some considerations should be made. First, all studies included populations of acutely ill patients with SMI, who needed hospitalization and were typically treated with polypharmacy regimens. The use of TDM as a surrogate marker for medication adherence in real-world acute settings requires further investigation, given the current lack of robust validation. Second, current literature on medication adherence is heterogeneous for design, clinical sample, and TDM methods. Three studies measured a single drug for TDM, each applying different criteria for drug selection (i.e. type of drug and its indication for psychiatric diagnosis) as well as for pharmacokinetic parameters (i.e. duration of treatment, dosing regimen, and interval since last administration). In our study, to improve the accuracy of the TDM estimation, we measured all medications in patients treated with polypharmacy, at steady state, considering the oral dose administered (DRRR), while excluding those with clinically significant drug–drug interactions. Moreover, to have a better estimation of drug adherence, we performed two independent clinical judgments at different time points (emergency room and in the psychiatric ward), while the other studies relied on a single measurement [patient self-report rating scales (Brasso *et al.*, 2021), emergency room clinical judgment (Lopez *et al.*, 2017)] or none at all (Geretsegger *et al*., 2019).

Our study has several strengths, including its naturalistic design and multiple assessment measures, a focus on acute inpatients with a wide range of SMI and polypharmacy regimens. Some limitations should be acknowledged. The observational single-center design can limit the generalizability; nonetheless, this approach increases the homogeneity of the estimations and the interrater reliability. The regression models had a poor fit, indicating that other factors may contribute to explaining the variability in adherence and agreement between measures; however, factors identified in our model can still be meaningful with weaker confidence. In addition, reasons for nonadherence (e.g. side effects, stigmatization) were not explored. We chose not to gather this information as the reliability of this information in an acute setting is questionable. Lastly, clinician assessments were dichotomous (adherent/nonadherent) and did not explore biases or explanations behind these conclusions. Although this method simplifies the complexity of adherence and may overlook certain specific aspects, it better represents the practical decision-making commonly applied in acute clinical settings. Future studies should examine the reasons behind clinicians’ judgment of adherence, including emotional and cognitive factors and potential stigma.

**Fig. 2:**
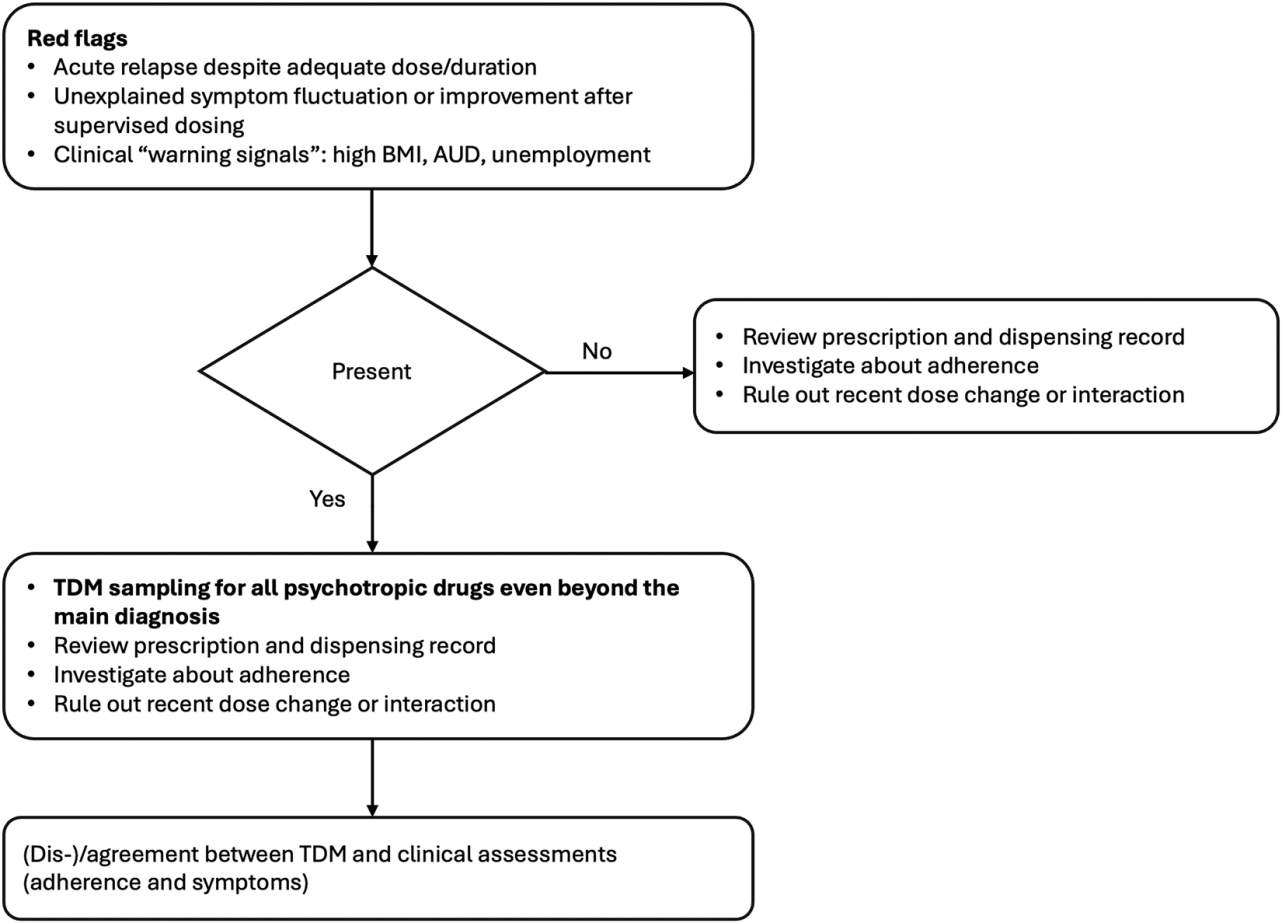
Algorithm for clinical decision-making that integrates TDM findings with clinical evaluation in the management of inpatients. When treatment nonadherence is suspected, clinicians should first look for red flags such as relapse patterns, symptom fluctuations, or risk factors (e.g. high BMI, alcohol use disorder, unemployment). If these factors are absent, the next step is to review prescriptions, dispensing logs, and adherence history. If red flags are present, TDM should be performed for all psychotropic medications, followed by a re-evaluation of adherence and clinical symptoms to assess the concordance between TDM results and the clinical presentation. AUD, alcohol use disorder; TDM, therapeutic drug monitoring.

Overall, our study highlights the limitations of relying solely on indirect measurements in the assessment of adherence and suggests its integration with direct methods to support clinical decision-making and guide pharmacological choices (Fig. 2); however, the financial and logistical feasibility of TDM assessments needs further investigation through prospective controlled trials.

## Acknowledgements

We are particularly grateful to the residents of the 2017–2019 cohorts at the School of Specialization in Psychiatry, University of Padova, whose invaluable support in data collection was instrumental to this work.

Conceptualization and supervision: G.P. and F.S. Methodology: G.P., F.S., C.A., N.M., T.T., and M.P. Investigation: G.P., F.S., M.P., and T.T. Formal analysis, visualization, and writing – original draft: N.M. Writing – review and editing: G.P., F.S., C.A., T.T., and M.P.

### Conflicts of interest

There are no conflicts of interest.
